# Unzipping hBN with ultrashort mid-infrared pulses

**DOI:** 10.1126/sciadv.adi3653

**Published:** 2024-05-01

**Authors:** Cecilia Y. Chen, Samuel L. Moore, Rishi Maiti, Jared S. Ginsberg, M. Mehdi Jadidi, Baichang Li, Sang Hoon Chae, Anjaly Rajendran, Gauri N. Patwardhan, Kenji Watanabe, Takashi Taniguchi, James Hone, D. N. Basov, Alexander L. Gaeta

**Affiliations:** ^1^ Department of Electrical Engineering, Columbia University, New York, NY 10027, USA.; ^2^ Department of Physics, Columbia University, New York, NY 10027, USA.; ^3^ Department of Applied Physics and Applied Mathematics, Columbia University, New York, NY 10027, USA.; ^4^ Department of Physics, Indian Institute of Technology Guwahati, Assam 781039, India.; ^5^ Department of Mechanical Engineering, Columbia University, New York, NY 10027, USA.; ^6^ School of Electrical and Electronic Engineering, Nanyang Technological University, Singapore 639798, Singapore.; ^7^ School of Materials Science and Engineering, Nanyang Technological University, Singapore 639798, Singapore.; ^8^ School of Applied and Engineering Physics, Cornell University, Ithaca, NY 14853, USA.; ^9^ Research Center for Functional Materials, National Institute for Materials Science, 1-1 Namiki, Tsukuba 305-0044, Japan.; ^10^ International Center for Materials Nanoarchitectonics, National Institute for Materials Science, 1-1 Namiki, Tsukuba 305-0044, Japan.

## Abstract

Manipulating the nanostructure of materials is critical for numerous applications in electronics, magnetics, and photonics. However, conventional methods such as lithography and laser writing require cleanroom facilities or leave residue. We describe an approach to creating atomically sharp line defects in hexagonal boron nitride (hBN) at room temperature by direct optical phonon excitation with a mid-infrared pulsed laser from free space. We term this phenomenon “unzipping” to describe the rapid formation and growth of a crack tens of nanometers wide from a point within the laser-driven region. Formation of these features is attributed to the large atomic displacement and high local bond strain produced by strongly driving the crystal at a natural resonance. This process occurs only via coherent phonon excitation and is highly sensitive to the relative orientation of the crystal axes and the laser polarization. Its cleanliness, directionality, and sharpness enable applications such as polariton cavities, phonon-wave coupling, and in situ flake cleaving.

## Teaser

Mid-infrared laser pulses mechanically vibrate atomic bonds in hBN to gently tear the sample along a well-defined direction.

## INTRODUCTION

Existing nanostructuring methods achieve nanometer-resolution features with cleanroom-assisted processes such as electron beam lithography (*1*–*4*) and etching (*2*–*4*) or in situ femtosecond laser writing (*5*). However, the former are time-intensive, are costly, and require multistep processing, and the latter relies on ablation. Both approaches leave residue or debris. Femtosecond laser writing, aided by multiphoton absorption and the generation of ionized electrons, is a thermal process that deposits heat locally in the region being excited and thus is not able to produce structures much smaller than the excitation wavelength (*6*). At sufficiently high excitation energy, the material will undergo structural changes such as burning or ablation patterns consistent with heat deposition (*6*). Our unzipping technique generates structures on the nanoscale, orders of magnitude below the mid-infrared (IR) diffraction limit, without ablation. Features are written in situ directly (resist-free) on hexagonal boron nitride (hBN) without vacuum or cryogenics in seconds, and the flake remains clean.

**Fig. 1.**
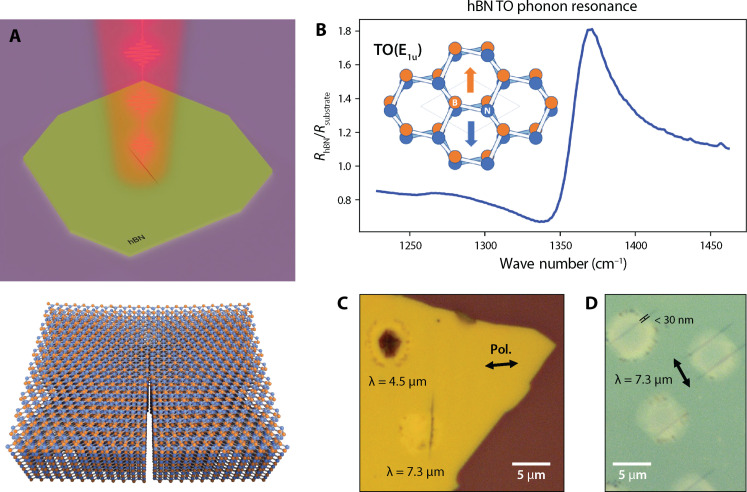
A phonon-resonant effect. “Unzipping” occurs only when hBN is strongly driven at its TO phonon resonance and yields ablation-free line defects. (**A**) Top: In this experiment, a pulsed mid-IR laser is focused onto an hBN flake, producing a localized edge or “zip.” Bottom: The zip is oriented along the armchair axis. (**B**) Fourier transform infrared (FTIR) spectroscopy linear reflectance spectrum about the hBN TO(E_1u_) phonon resonance on pristine hBN relative to the SiO_2_/Si substrate, centered at 1367 cm^−1^ (λ = 7.3 μm). Inset: AA′-stacked hBN illustrating the mode of interest. We directly drive this mode with a laser tuned to 7.3 μm to subject the crystal to high in-plane lattice-scale strain. (**C**) Comparison of off- and on-resonant ultrafast irradiation of a 70-nm flake at λ = 4.5 and 7.3 μm, respectively. The latter results in a highly sub-wavelength line defect (zip); burning is absent, and the line is oriented roughly perpendicular to the laser polarization. Off-resonant irradiation at 4.5 μm generates a wavelength-scale burned spot and lacks polarization dependence. (**D**) A series of clean, parallel zips produced by a perpendicularly polarized laser on a single flake with height of 38 nm. The width here measures <30 nm (fig. S6).

The in-plane hexagonal crystal structure of many two-dimensional (2D) van der Waals (vdW) materials yields two high-symmetry axes separated by 30°, known as zigzag and armchair. In hBN, the TO(E_1u_) phonon at 7.3 μm corresponds to in-plane atomic motion parallel to the zigzag axis (Fig. 1B, inset) where boron and nitrogen displace in opposite directions. This optical phonon is dipole active and lies in the laser-accessible mid-IR regime due to the material’s light constituent atoms. Coherent resonant excitation of the phonon at pulse intensities of 10 TW/cm^2^, within the linear phonon-driving regime and far below the estimated laser-induced damage threshold of 50 TW/cm^2^ (*7*, *8*), was calculated to yield transient strains corresponding to atomic displacements of 10% of the equilibrium lattice constant (*7*). Furthermore, nonlinear phononics has been demonstrated to dynamically modify the symmetry of materials (*9*–*13*), resulting in structural phase transitions and ferroic behavior. Preferential orientation of flake fracture and crack formation and propagation has been studied previously in hexagonal vdW materials subject to exfoliation forces (*14*). Predictably, the histograms cluster around the armchair and zigzag axes, with an angular spread about each orientation. Graphene and hBN have roughly equal preference for armchair and zigzag, while 2H-MoS_2_ and analogous transition-metal dichalcogenides display strong directional preference (*14*, *15*).

In this study, we take an approach to deterministically inducing flake fracture by exploiting the natural vibration of atoms and driving them coherently with mid-IR pulses. Here, pulse intensities of 50 to 65 TW/cm^2^ generate larger atomic displacements than previously studied and lead to macroscopic material structuring in the form of controllable, localized, rapid crack propagation. These displacements amplify lattice-scale strain and prompt organized fracture along an imposed symmetry. This approach allows us to introduce atomic-scale line defects to ultrahigh-purity exfoliated hBN flakes by directly accessing the TO(E_1u_) mode from free space, which we demonstrate in samples with height of 24 to 76 nm. The features are seeded from a random point within the laser-driven spot, possibly originating from an intrinsic defect, and unzip from the tip with largely unidirectional growth. The speed of formation is estimated to be on the order of 100 μm/s. This work represents the demonstration of optically generated single-line defects using a wavelength far below the material bandgap, and this unzipping phenomenon occurs only through coherent driving of hBN.

## RESULTS

### Characteristics of unzipping

The experimental setup in which we gate the high-intensity pulses using a manual shutter until the damage threshold is reached is illustrated in fig. S1. In Fig. 1C, a tunable femtosecond source irradiated a single flake at neighboring spots, both on (λ = 7.3 μm) and away from (λ = 4.5 μm) the hBN optical phonon resonance. The source polarization was identical in both cases, and irradiation continued until the first indication of structural change to the flake. We observe several critical differences in the sample at the two excitation wavelengths. A line defect appears at the on-resonant spot, while the off-resonant spot is burned and ablated. The minimum achievable zip width is measured to be <30 nm despite being generated with a λ = 7.3 μm beam, making the features highly sub-wavelength in scale. With off-resonant irradiation, the size of the ablated spot is on the order of the irradiation wavelength, as expected for conventional laser damage via localized thermal heating. Line defects do not appear at the off-resonance damage threshold even with the same polarization and flake orientation, further confirming that unzipping is associated with on-resonance driving. The absence of ablation, seen more clearly among several repeated zips on a single flake in Fig. 1D, distinguishes unzipping as a method of gentle and wavelength-selective defect creation.

**Fig. 2.**
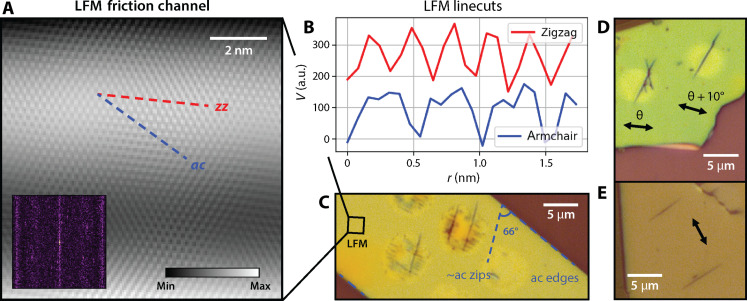
Polarization dependence. Unzipping is sensitive to the pump laser polarization. (**A** to **C**) Atomic-scale lateral force microscopy (LFM) determines the crystal orientation of an hBN flake and hence the unzipping direction. The scan region is marked in (C). (A) LFM friction channel image after filtering, with zigzag and armchair directions marked. Inset: 2D fast Fourier transform (FFT) of unfiltered friction channel. (B) Linecuts along the zigzag and armchair directions yield periodicities of 29 and 50 Å, respectively, confirming the measurement. The *y* axes are offset for clarity. a.u., arbitrary units. (C) The 70-nm-thick flake imaged with LFM. The zips measure 66° from the armchair-oriented flake edges, making them nearly parallel to an armchair axis of the crystal. (**D** and **E**) The unzipping phenomenon occurs independently of the choice of substrate. (D) Unzipped lines on a 60-nm-thick hBN flake on SiO_2_/Si. The sensitivity of unzipping to pump laser polarization is evident in the transition from an X-shaped line defect to a parallel single line under a 10° shift in polarization. (E) Unzipped lines on an 84-nm-thick hBN sample on sapphire. Zips generated by the same polarization are parallel.

**Fig. 3.**
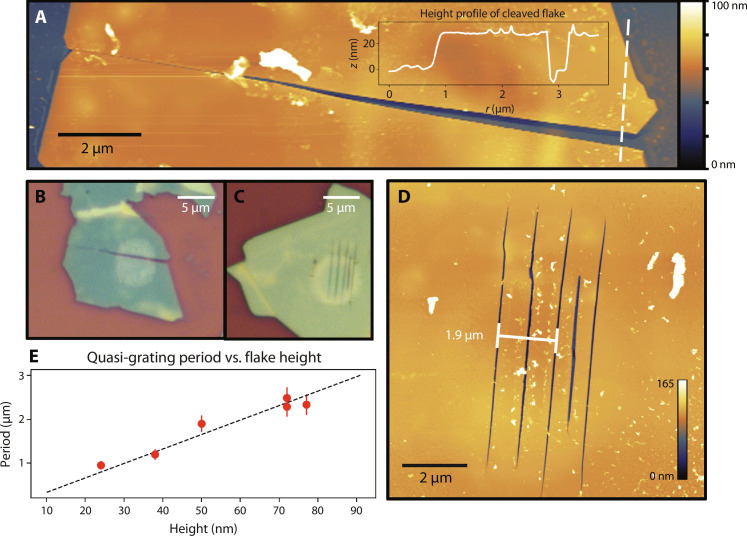
Applications in flake patterning and nanostructuring. All-optical in situ patterning of hBN flakes using the mid-IR phonon-resonant technique. (**A** and **B**) Unzipping can be controllably and boundlessly extended once initiated, achieving an ultrahigh aspect ratio. (A) AFM topography: A 24-nm-thick flake is cleaved in two by extending an initial unzipped line in both directions. A slight edge offset at the left and right ends of the cleavage line reveal that the bottom section has rotated and shifted along the substrate. Inset: Height profile along the dashed linecut confirms full separation of the cleaved sections. (B) Micrograph: The initial unzipped line is localized within the slight discolored spot. (**C** and **D**) Quasi-periodic gratings generated by the unzipping technique on a 50-nm-thick flake, represented in a micrograph (C) and topographic AFM image (D). (**E**) Linear trend between grating period and flake height. Error bars indicate the range of periodicities displayed by the quasi-periodic gratings.

**Fig. 4.**
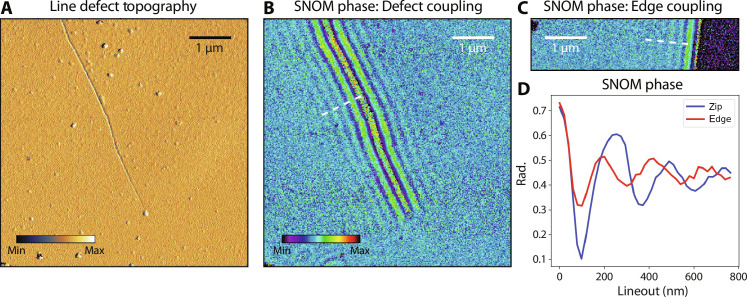
Atomically sharp edges. Unzipped lines are atomically sharp and exhibit highly efficient coupling to phonon-polaritons. This flake is 38 nm in height. (**A**) AFM topography of a kinked (suboptimal) zip. (**B** and **C**) Near-field phase images under 1494.5 cm^−1^ excitation, as probed by scattering-type scanning near-field optical microscopy (s-SNOM). (B) Phonon-polariton coupling via an unzipped line. (C) Coupling via a natural flake edge formed by mechanical exfoliation. (**D**) Unzipped lines are atomically sharp and can outperform flake edges in coupling to phonon-polaritons, as evidenced by a comparison of modulation depth and decay rate along the dashed linecuts in (B) and (C).

Because unzipping is the product of selective phonon driving, it inherits symmetries from the crystal. As expected for a system with rotational symmetry, the ease of unzipping hBN depends on the relative angle between the laser polarization and the crystal axes. Unzipped lines form roughly perpendicular to the polarization, with a deviation of ±15°. For a given flake orientation, not every polarization can produce a line defect. Sweeping all relative angles between the sample orientation and pump polarization, as shown in fig. S2, revealed the expected sixfold symmetry. The distribution of these polarizations is periodic: the sector width of polarizations that unzip hBN is ≤50° and repeats every 60°. Polarizations falling outside of these ranges will produce a dense crosshatch pattern (fig. S2D). Nevertheless, in contrast to the off-resonant example, the structural changes retain a straight-edged, ordered geometry. Furthermore, the optimal polarization within each sector yielding the highest-quality unzipped lines that are atomically sharp, clean, and straight is parallel to the zigzag axis or perpendicular to the resulting zip. (This property can also be used to determine an unknown flake orientation.) The farther the polarization strays from optimum, the more resistant the flake is to unzipping. The sample would require prolonged laser exposure and exhibit slower crack propagation with increased likelihood that the lines will be wider, kinked (examples in Figs. 2D, 3D, and 4A), feathered (example in Fig. 2D), or produce light debris (examples in Fig. 1C and fig. S2A). If the laser polarization is not optimized relative to the crystal orientation, then the lines formed will not be perfectly straight, instead exhibiting these kinks as a result of branching between several parallel armchair axes to satisfy the local energy conditions. These zips also appear wider.

Regardless of zip quality, all line defects created via phonon-resonant excitation are sharply tapered at the ends, which supports the picture of unzipping as originating from bond rupture. Driving the TO(E_1u_) phonon stretches bonds parallel to the zigzag axis, which we expect leads to fracture primarily in the direction perpendicular to the applied strain or along the armchair axis. To test this hypothesis, we performed atomic-scale lateral force microscopy (LFM) (*16*, *17*) on our exfoliated flakes to determine the crystal orientation, because the standard second-harmonic generation (SHG) method is unsuitable in hBN beyond the few-layer limit (*18*–*20*). Figure 2A displays the filtered friction channel image with its raw 2D fast Fourier transform (FFT). Figure 2B shows the friction channel linecuts along the zigzag (zz) and armchair (ac) directions. Periodicities were measured to be *T*_zz_ = 2.9 Å and *T*_ac_ = 5.0 Å, respectively. The ratio *T*_ac_/*T*_zz_ is equal to √3 as expected for a hexagonal lattice, despite a proportional deviation from the accepted hBN lattice periodicities of 2.5 and 4.3 Å (*21*). From the LFM measurements, we conclude that the flake in Fig. 2C unzips approximately along the armchair direction (66° from the armchair flake edge) even when created under suboptimal conditions. While the overall unzipping angle may be slightly off from 60°, the constituent segments of the kinked zip strongly prefer to fall along the armchair axis.

In addition to single unzipped lines, there are variations on unzipping that can appear under comparable irradiation conditions. As seen in Figs. 1D and 2C, two independent yet parallel lines may form simultaneously. Figure 2D displays an example of the rarer X-shaped unzipping due to its high sensitivity to relative polarization. Two kinked, near-armchair zips separated by ~50° result from symmetric activation. While the individual zips are no longer perpendicular to the driving polarization (as in the standard case), their angle bisector is, and shifting the polarization by just 10° recovers a single zip parallel to one of them. Atomic-scale line defects have also been generated in monolayer graphene using electron beam irradiation (*15*), but, unlike the unzipping phenomenon, they exhibit a slower crack propagation speed of 1 μm/s and lack polarization dependence or X-shaped features. This contrast reaffirms the role of laser-phonon driving in our experiment.

An added feature of the unzipping technique is that it is consistent and robust. For a given flake at fixed orientation driven by the same allowed pump polarization, all zips formed will be parallel. This is illustrated in Figs. 1D and 2E. Furthermore, these line features can be generated in flakes of various thickness spanning tens of nanometers, even with a moderately misaligned laser, slightly irregular beam shape, variation in average pump intensity and pulse-gating pattern, and/or suboptimal pump polarization. We also refer to fig. S2, which illustrates three categories of unzipping: “proper,” suboptimal, and failed zips. Proper unzipping is characterized by clean lines and an absence of ablation damage, produced when the laser polarization is perpendicular to the armchair crystal orientation. Figure 2C shows an example of a flake that unzipped along the armchair axis despite poorer beam shape and alignment, which resulted in a zip that was not as sharp, clean, and straight. [The irregular streaking within these irradiated spots is not unzipping, and the flake appears smooth under atomic force microscopy (AFM). See fig. S3B.]

Last, the unzipping effect itself occurs independently of the substrate. Features were produced primarily on flakes on SiO_2_/Si, but unzipped lines also form on hBN on sapphire (Fig. 2E). [We note that the slight discolored spots are a substrate effect in silicon (*22*) and are not seen on samples exfoliated onto sapphire (Fig. 2E). Discoloration is present on the irradiated bare substrate in fig. S4 regardless of mid-IR wavelength.] However, unzipping flakes of similar thickness on sapphire is more difficult due to poorer flake adhesion, resulting in greater sensitivity to the irradiation parameters. These flakes tend to unzip and immediately rip or peel from the substrate. To combat the less robust nature of unzipping on sapphire, we reduced the average pump intensity by a factor of 3 and operated within a narrower viable fluence window.

### Applications of unzipping

One application of unzipping is an all-optical, orientation-selective, in situ method for cleaving or patterning flakes. In Fig. 3 (A and B), unzipping cuts completely through thinner <30-nm-thick flakes. Once an unzipped region is created (localized within the faint circle), it can be easily and boundlessly elongated by shifting the pump beam incrementally along the unzipping axis with a piezoelectric stage. Even without a stage, the line can be extended cleanly by irradiating slightly beyond its existing endpoints. This technique may be used to cleave a single hBN flake for self-oriented stacking in a moiré homostructure (*23*). Similarly, adjacent regions can be irradiated with different polarizations, and the resulting zips will snap together at 60° angles (fig. S5), opening the door to custom flake shapes and linear feature designs. These examples illustrate hBN’s potential as a patterned, orientation-selective layer, either by itself, as a mask (*2*), or in a 2D heterostructure (*24*), especially because it is mechanically tough (*25*), has a large bandgap (*26*), and is widely used as an encapsulating material (*27*).

The unzipping mechanism can also generate quasi-periodic gratings (Fig. 3, C to E) at slightly higher irradiation fluences. These gratings emanate from an initial unzipped line, mainly in one direction, at a speed of 10 μm/s. In some cases, they can be introduced separately by irradiating an existing unzipped region. We hypothesize that these gratings form when intense phonon-polaritons (*28*) are launched from the initial unzipped region, leading to a “rippling” through the crystal (the resulting surface is not raised). However, an investigation into the exact mechanism is beyond the scope of this work. The grating period follows a linear trend with flake height (Fig. 3E), where we define the grating period as twice the line spacing to correlate the periodicity with the phonon-polariton wavelength. Notably, unzipping and their quasi-periodic gratings are fundamentally distinct from nanogratings (*29*) and laser-induced periodic surface structures (*30*, *31*). The nanostructures described here display atomically sharp, tapered lines with a low-duty cycle, are associated with a resonant phonon effect instead of plasma formation, and are oriented along the armchair crystal axes. Quasi-periodic gratings may find applications in enhancing wave coupling from free space or as a standalone nanopatterning technique.

Figure 4 demonstrates efficient coupling from free-space optical excitation to polaritons in hBN via unzipped nanostructures. Figure 4D compares linecuts of scattering-type scanning near-field optical microscopy (s-SNOM) phase images from excitation within the hBN upper Reststrahlen band: Phonon-polaritons launched by the unzipped line exhibit much greater modulation depth and slower decay than those by a natural edge on the same flake. We conclude that unzipped “artificial edges” can outperform exfoliated edges in coupling to polaritons and give the user freedom of edge placement. Compared to conventional coupling methods (*32*), unzipping yields a directional phase front, as opposed to launching from an AFM tip (*28*, *33*), and is a quick, cleanroom-free technique, unlike electron beam patterning (*32*) or gold nanostructures (*32*, *34*).

**Fig. 5.**
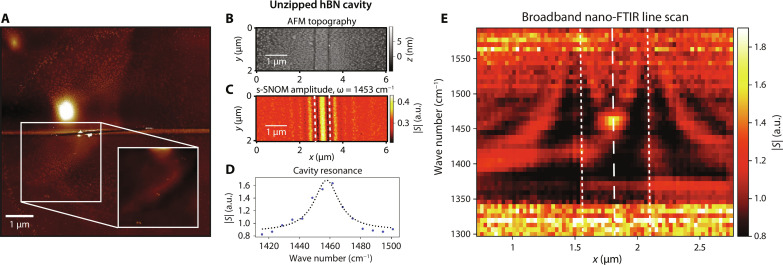
Polariton cavity and defect seeding. (**A**) AFM topography of a 50-nm-thick flake subject to nanoindentation patterning. The flake was then irradiated on resonance until an unzipped line sprouted from a defect-seeded location. Inset: A unique nanoindented pattern on the flake, before irradiation at λ = 7.3 μm, written for localized identification. (**B** to **E**) A Fabry-Perot polariton cavity in hBN fabricated by the unzipping technique, imaged by near-field techniques. This flake is 40 nm in height and features a 630-nm-wide cavity. (B) AFM topography of the unzipped hBN cavity. (C) s-SNOM amplitude image of the cavity probed near resonance at 1453 cm^−1^ shows field confinement. The cavity boundaries are marked by dotted white lines. (D) Cavity resonance lineshape with an estimated *Q* ≈ 70 extracted from a Lorentzian fit to the data points. Its corresponding linecut in the hyperspectral image (E) is marked by the dashed white line. (E) A hyperspectral line scan across the cavity obtained via nano-FTIR. The cavity boundaries are marked by dotted white lines.

We also show that the location of unzipped lines can be deterministically controlled by defect seeding via nanoindentation (Fig. 5A). A distinct pattern of nanoindents, seen in Fig. 5A inset, was written on the flake to aid in identifying the area of interest. We then irradiate the flake with phonon-resonant pulses at half the power used in the standard unzipping procedure until a zip sprouts from one of the nanoindents. Most notably, the non-nanoindented areas of the flake were insensitive to the reduced laser power at resonance even after 10 s of total exposure time, whereas the nanoindented regions unzipped after a fraction of a second. No line was generated on the pristine sample at equal power because its threshold for unzipping is much higher. Consequently, nanoindentation provides a way to spatially localize unzipping on demand.

A demonstration of unzipping’s capacity to yield potentially useful devices is the realization of a Fabry-Perot polariton cavity. We show that small cavities <1 μm in width can be created with the mid-IR laser source, and Fig. 5B shows the AFM topography of such a structure, where a 630-nm-wide slab of hBN is cut from the surrounding flake by the unzipping technique. Figure 5C displays an s-SNOM scattering amplitude image of the unzipped cavity taken at ω = 1453 cm^−1^, following calculated dispersion relations for hyperbolic phonon-polaritons (HPhPs) in hBN by Dai *et al.* (*28*). The high field amplitude seen in the center of the cavity agrees with the more exhaustive broadband nano–Fourier transform infrared (FTIR) line scans across the cavity that reveal a resonance peak around 1457 cm^−1^ (Fig. 5, D and E). After a linear background is subtracted from the data and the peak is fitted to a Lorentzian lineshape, we extract a quality factor *Q* = ω_0_/Δω = 1457 cm^−1^/20.6 cm^−1 ^≈ 70, with an SD of 2.3 cm^−1^ in the full width at half maximum (FWHM) fit.

Our unzipped cavity achieves comparable performance to similar all-hBN planar resonators fabricated by well-established methods. Structures created via electron beam lithography and etching (*35*, *36*) have demonstrated *Q* factors between 50 and 100. We believe the atomically sharp edges characteristic of unzipping provide an advantage over etched edges. In addition, these cleanroom-fabricated structures have a more optimal resonator geometry, hBN slabs bounded by air, while our cavities are bordered by narrow trenches several tens of nanometers across. Improvements in the geometry of our preliminary unzipped cavity device could yield stronger field enhancement for applications in light-matter interaction.

## DISCUSSION

We have characterized the unzipping phenomenon as fundamentally dependent on the wavelength of the driving laser and its polarization relative to the crystal axes, and the results of the nanoindentation experiment confirm that it can be seeded by macroscopic defects placed wherever desired by the user. To explore the underlying principles of the effect, we performed the unzipping procedure on a flake exfoliated from a commercially available hBN crystal. Compared to the ultrahigh-quality flakes studied in the rest of this work, the lower quality commercial samples on the same substrate unzipped with lower irradiation power and substantially reduced laser exposure time. Furthermore, we could easily and cleanly unzip thicker ~100-nm flakes by slightly increasing the laser power, whereas we were not able to do this on the ultrahigh-quality sample. These observations strongly suggest that unzipping is also seeded by defects at the atomic level.

Existing literature on bulk and 2D material fracture mechanisms offer some possible clues to explain the unzipping phenomenon. In the case of nanoindentation, macroscopic defects function as “pre-cracks” that prime crack growth and propagation following the classical Griffith theory of brittle fracture (*37*, *38*). They are known to dominate nanoscale defects in determining crack behavior. Crack propagation and the effect of microscopic defects in 2D materials on the atomic scale are less well understood. One study showed that the placement of atomic dislocations near the crack tip zone in MoS_2_ influenced the path of the propagating crack by modifying the strain field directly in front of the tip (*39*). As the crack advanced with each snapshot in time, the dislocation would appear again at a different location in front of the tip, influencing the next advancement and so on. This “dislocation emission” theory at the nanoscale goes beyond traditional brittle fracture theory. We hypothesize that coherent resonant driving at the phonon resonance breaks atomic bonds, preferentially near intrinsic defects in the crystal, seeding and growing nanoscale defects until they are large enough to qualify as macroscopic pre-cracks governed by classical fracture mechanisms. Past this threshold, catastrophic rupture characteristic of the Griffith model overtakes and leads to the observable unzipping motion. According to time-dependent density functional theory simulations, irradiating hBN at the TO(E_1u_) phonon frequency at substantially lower optical powers than are used for the unzipping technique already produces atomic displacements nearing 10% of the lattice constant (*7*). At fivefold greater pulse intensities of >50 TW/cm^2^ here, nonlinear effects arise and higher bond strain is induced. We hope future theory calculations will illuminate the complex mesoscopic dynamics underlying the mechanical fracturing generated by optical excitation.

In summary, strong resonant driving of hBN at its 7.3 μm TO(E_1u_) phonon resonance allows us to unzip the flake or place atomically sharp edges within the flake interior, along the armchair crystal direction. In contrast to the flake burning typical of strong off-resonant irradiation, this gentle and debris-free technique of selectively driving a vibrational mode generates localized, directional bond strain within the material. The result demonstrates optically induced strain strong enough to cause flake fracture. This unzipped region can be extended infinitely along the flake for in situ orientation-selective cleaving. We also show that, while unzipping is driven by a mid-IR laser with a correspondingly large beam size, it directly fabricates lines and structures on the nanoscale. The sharp edges generated by unzipping hBN exhibit highly efficient coupling to phonon-polaritons and can confine mid-IR HPhPs to sub-micrometer length scales in a Fabry-Perot cavity with *Q* ≈ 70. Our technique may be generalized to other polar crystals with IR-active optical phonons, such as SiC or α-MoO_3_, which have phonon modes around 10 μm (*40*–*42*). The unzipping phenomenon is fundamentally elegant yet also offers practical purpose in localizing and enhancing mid-IR emission, mask patterning, custom flake shaping, and molecular sensing (*36*).

## MATERIALS AND METHODS

### Sample preparation

Ultrahigh-quality AA′-stacked hBN (intrinsic defect density 10^9^ cm^−2^) is mechanically exfoliated using low-residue Scotch Magic Greener tape onto 285-nm SiO_2_-on-Si and sapphire substrates that were first subject to an O_2_ plasma treatment to remove adsorbates. No annealing was performed after exfoliation.

A commercially available hBN crystal from HQ Graphene was exfoliated onto a SiO_2_-on-Si substrate for a comparison of unzipping performance.

### Phonon-resonant irradiation of hBN

The hBN flakes are irradiated with a 1-kHz pulsed mid-IR laser tuned to the hBN TO(E_1u_) phonon at 1367 cm^−1^ (λ = 7.3 μm). We achieve this output with a laser system consisting of a Ti:sapphire mode-locked oscillator (KMLabs Griffin), Ti:sapphire chirped pulse amplifier with regenerative amplification outputting 6-mJ pulse energy at 1-kHz repetition rate (Coherent Legend Elite), optical parametric amplifier (Light Conversion HE-TOPAS Prime), and a subsequent difference-frequency generation module (Light Conversion NDFG). The resulting mid-IR pulses measure 120 fs in pulse width and 1.5-μm FWHM.

The beam is routed to the sample in reflection geometry at normal incidence with a mid-IR dichroic mirror (ISP Optics BSP-DI-25-3) and focused with a 40× reflective objective (Thorlabs LMM-40X-P01, 0.5 numerical aperture). The incident average power is 250 μW. Pulse selection is performed manually with an electronic shutter (Melles Griot) as we monitor the formation of unzipped lines on a charge-coupled device camera mounted in the reflection path. The shutter speed is tunable from ^1^/_60_ to 2 s, although successful unzipping is achieved with the shutter operating at ^1^/_4_ second or less. The total laser fluence required to achieve an initial unzipped line defect is on the order of 10^3^ J/cm^2^ (variable, depending on relative polarization and local flake thickness and uniformity), adjusted with a combination of ZnSe reflective neutral density (ND) filters (Thorlabs), mid-IR polarization optics, pulse gating settings (shutter speed and repetitions), and an optional band-pass filter at 7500 ± 50 nm (Thorlabs). hBN on SiO_2_/Si unzipped with an average pulse intensity of 50 TW/cm^2^; flakes on sapphire required lower peak power due to weak substrate adhesion. All defect creation was performed under ambient conditions.

The angle between the laser polarization and crystal orientation was controlled by either rotating the sample relative to a fixed linear polarization or adjusting the pump polarization using mid-IR polarization optics (Alphalas tunable zero-order waveplates, Thorlabs ZnSe wire grid polarizers), which yielded equivalent results. Extending the unzipped line requires substantial pump power reduction that we achieve with the 7.5-μm band-pass filter (Thorlabs). It cuts the total power by 85% while still maintaining a spectrum that overlaps the transverse optical (TO) phonon resonance in Fig. 1B. This can also be substituted for additional ND filters. The most uniform line defect extension is achieved by incrementally translating the sample on a piezoelectric stage. Another technique is to irradiate an adjoining spot; the independently generated unzipped lines will merge.

### Off-resonant irradiation of hBN

To confirm that unzipping is a resonant effect, we repeat the defect generation process at an off-resonant mid-IR wavelength. The experimental setup is identical, with the output of the NDFG module now tuned to λ = 4.5 μm (τ*_p_* = 100 fs, 1-μm FWHM). The fluence required to straddle the damage threshold here is very similar to that of the resonant case (at minimum, the same order of magnitude).

### AFM topographic imaging

AFM topography was imaged on a Bruker Dimension Icon in automated tapping (ScanAsyst) mode with ScanAsyst-Air probes at 0.5-Hz scan rate and 128-pixel resolution. Plane leveling and post-processing were done in Gwyddion (*43*).

### Atomic-scale imaging of hBN crystal orientation

We determine the crystal orientation of hBN flakes using atomic-scale LFM. Friction channel scans were taken on a Bruker Dimension Icon with a silicon nitride tip on a silicon nitride cantilever (Bruker DNP-C, nominal *k* = 0.24 N/m) at 2.5-Hz scan rate in constant height mode. Residual noise from the XY sensors can affect scans at the angstrom level, so the XY closed-loop control parameter should be turned off. A fresh antistatic ionizing cartridge containing alpha particle-emitting polonium-210 (StaticMaster CPSMR3) was placed near the sample to neutralize electrostatic effects impeding tip engagement and imaging. We then performed plane leveling and 2D FFT filtering on the raw images in Gwyddion.

### Nanoindentation

Nanoindents were created on hBN flakes to controllably localize and seed zip creation. The procedure, similar to that of an experiment where nanoindentation was used to write quantum emitters in hBN (*44*), was performed on a Bruker Dimension Icon in the PeakForce quantitative nanomechanical mapping workspace in contact mode. We chose BudgetSensors Tap300DLC (measured *k* = 101 N/m; 15-nm tip radius), a silicon AFM tip with a diamond-like carbon coating, for durability on a hard material like hBN. Nanoindentation patterns were generated using Point and Shoot under the Ramp menu. The deflection trigger threshold, or maximum force applied to the sample, was set to a value between 21 and 28 μN where the force curve first exhibited signs of plastic deformation. The X Rotate parameter was assigned as 20° to minimize lateral plowing of the surface by the tip, which is prone to pitching forward during the indentation phase. A topographical scan of the nanoindented sample was then imaged using the same tip.

### Scattering-type scanning near-field optical microscopy

Near-field images were obtained on a Neaspec GmbH neaSCOPE system equipped with a PtIr-coated AFM tip (ARROW-EFM, 20-nm tip radius) operating at 285-kHz tapping frequency. We use a broadly tunable light source (Daylight Solutions MIRcat quantum cascade laser) chosen to coincide in frequency with the hBN upper Reststrahlen band (1368 to 1610 cm^−1^) (*28*); the signal scattered off the probe tip is sent to a mercury-cadmium-telluride detector. We interferometrically detect and demodulate the signal at harmonics of the tapping frequency via the interferometric pseudo-heterodyne technique (*45*) to obtain background-free near-field signals with amplitude and phase information. The resulting images demonstrating phonon-polariton propagation in the vicinity of the hBN zip and a natural edge were then processed in Gwyddion. Topography information was obtained concurrently.

### Nano-FTIR spectroscopy

The cavity behavior in the near-field is probed by broadband nano-FTIR. The mid-IR light source was provided by broadband difference frequency generation from Light Conversion Orpheus Twins seeded by Light Conversion Pharos at 750-kHz repetition rate. The spectrum is detected via FTIR technique, and the signal is demodulated at harmonics of the tip tapping frequency. Multiple (~50 to 100) hyperspectral line scans across the cavity are averaged together.

### FTIR spectroscopy spectra

The far-field FTIR reflectance spectrum of pristine hBN was recorded with a Bruker Vertex 80v FTIR spectrometer coupled to a Bruker Hyperion II microscope. The aperture of the mid-IR illumination lamp was set to 25 μm. Spectra were acquired with 1000 averages and resolution of 2 cm^−1^. The SiO_2_/Si substrate was used as a reference.

### Scanning electron microscopy

Scanning electron microscopy (SEM) imaging was carried out using a Zeiss Sigma VP SEM. To avoid an electron charging effect, the accelerating voltage was set to 1 kV.
