# Supplementary material for: Unzipping hBN with ultrashort mid-infrared pulses
Source: Sci Adv. 2024 May 1;10(18):eadi3653. doi: 10.1126/sciadv.adi3653 (PMC11062566; doi:10.1126/sciadv.adi3653)
Supplement: Supplementary file 1 — Supplementary Text Figs. S1 to S6 [file sciadv.adi3653_sm.pdf]

Science Advances
AAAS

Supplementary Materials for

Unzipping hBN with ultrashort mid-infrared pulses

Cecilia Y. Chen *et al.*

Corresponding author: Alexander L. Gaeta, a.gaeta@columbia.edu

Sci. Adv. **10**, eadi3653 (2024)
DOI: 10.1126/sciadv.adi3653

This PDF file includes:

Supplementary Text
Figs. S1 to S6

Supplementary Text

From lines to shapes: Connecting zips at 60° angles

We can exploit the sensitivity of unzipping to the six-fold symmetry of the hBN crystal to write multiple zips within a region oriented integer-multiples of 60° apart. This extends the user-controlled method of introducing simple edges on the interior of existing flakes into a way to generate sharp vertices, or even to cleave customized flake shapes via unzipping. We predict the latter to be able to create polygonal flakes possessing sharp, armchair-oriented flake edges with edge lengths controllable to the sub-nm scale.

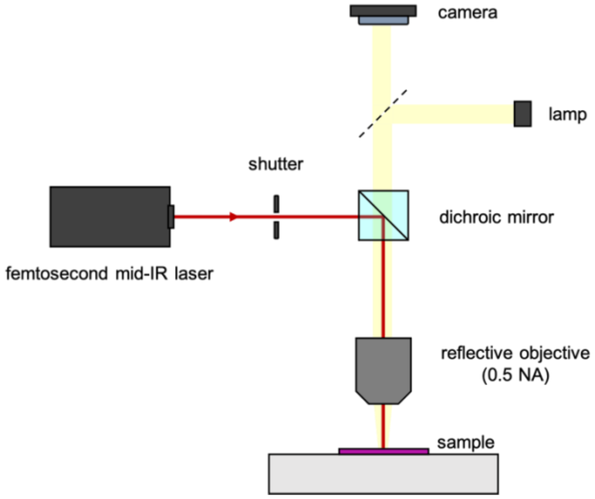

Fig. S1. Phonon-resonant irradiation setup for unzipping hBN.
Experimental setup for pulsed mid-IR excitation from free space.

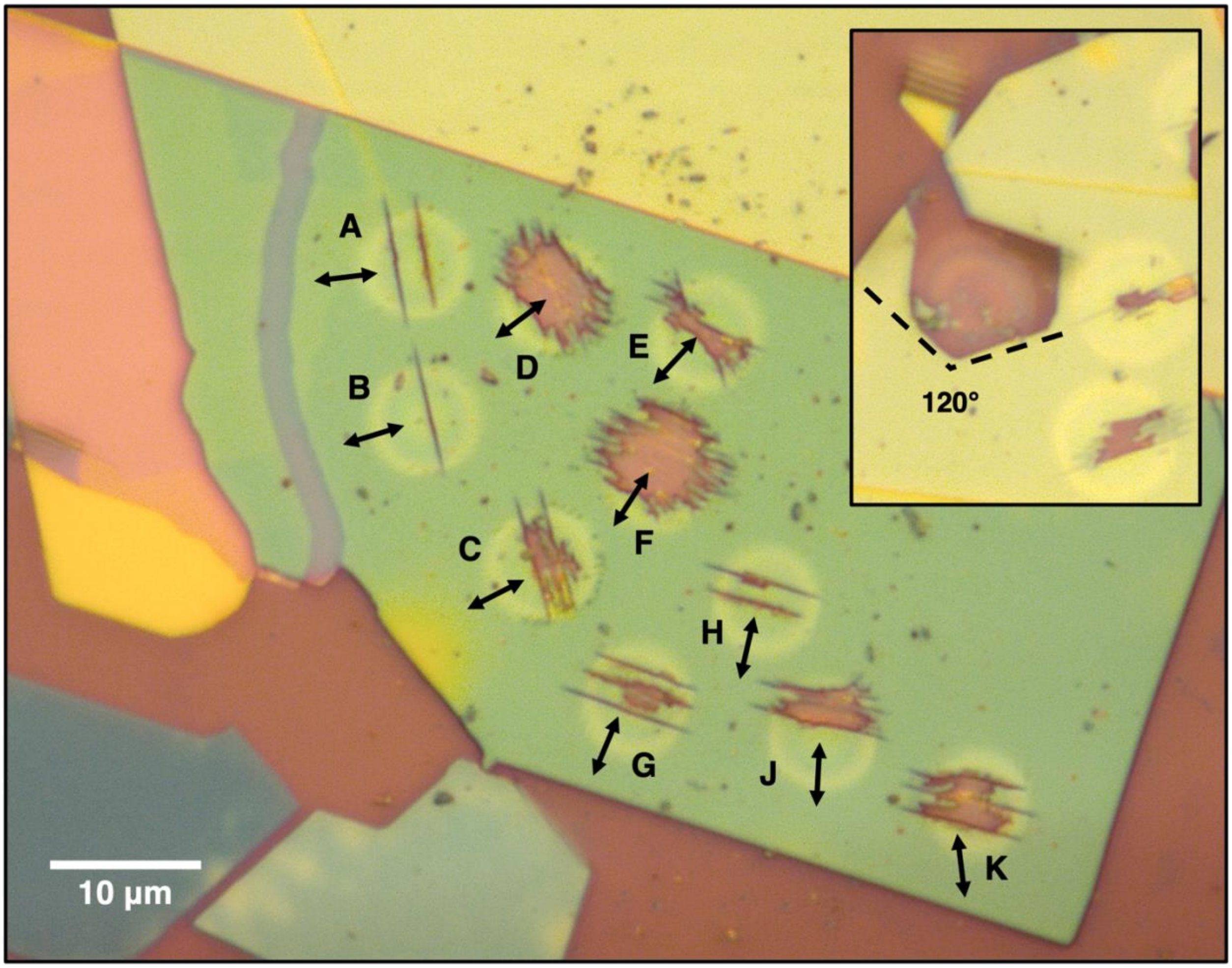

Fig. S2. Variation of features from strong coherent phonon driving: Proper versus failed unzipping.

A comparison of proper vs. failed unzipping due to different relative pump polarizations and fluences. The polarization is rotated through 90° in increments of 10° across spots **A-K**. The debris is from failed and suboptimal zips; proper zips are debris-free. Spots **A-B** are considered proper zips formed under the correct laser polarizations relative to the crystal orientation and within the acceptable fluence window, albeit with B more ideal than A; spot **C**, with lines along the same unzipping axis, was subject to both a less optimal polarization and a slightly higher fluence. Spots **G-K** unzipped (sub-optimally, due to excessive fluence) along an adjacent unzipping axis 60° away from the previous one. Crosshatched patterns in spots **D-F** result from polarizations that do not support unzipping. However, the constituent lines within the crosshatches are roughly parallel to the flake's preferred unzipping directions. **(inset)** Resonant irradiation in pursuit of unzipping (at any pump polarization) sometimes unpredictably ejects a polygonal piece of the flake, exposing the substrate. The resulting void possesses edges that follow the flake's unzipping directions (visible to the right), and at least one of its angles measures ~120°. We hypothesize that the unpredictability is due to local flake conditions on the macro- and nano-scale, such as flake delamination or non-uniformity, mechanical weakness near edges, or a higher concentration of intrinsic defects.

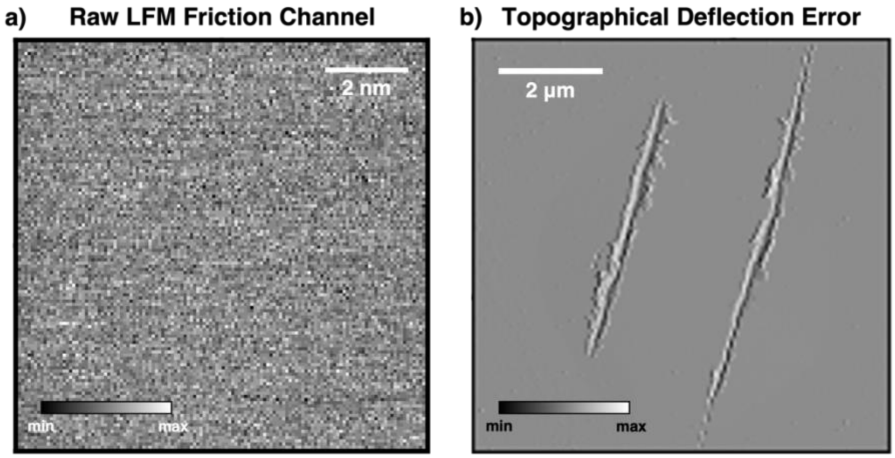

Fig. S3. Lateral force microscopy (LFM) and atomic force microscopy (AFM).

(A) Raw friction channel scan corresponding to Figs. 2A-C, with the AFM in atomic-scale LFM mode. **(B)** Topographical deflection error image corresponding to the double-unzipped spot in Fig. 2C. The flake surface remains smooth, even after unzipping with suboptimal irradiation conditions.

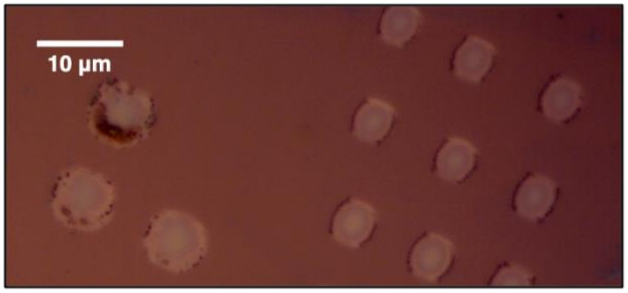

Fig. S4. Substrate discoloration in silicon.

Discolored spots in unzipped hBN samples are present only on SiO_2/Si substrates and absent in flakes processed on sapphire substrates (Fig. 2E). This discoloration effect is independent of the hBN and appears when irradiating the silicon-based substrate at various mid-infrared wavelengths(*22*). The larger circles on the left correspond to irradiation at λ = 7.3 µm; the smaller circles on the right correspond to λ = 4.5 µm. The size of the discoloration footprint as the beam is focused onto the sample scales with the wavelength.

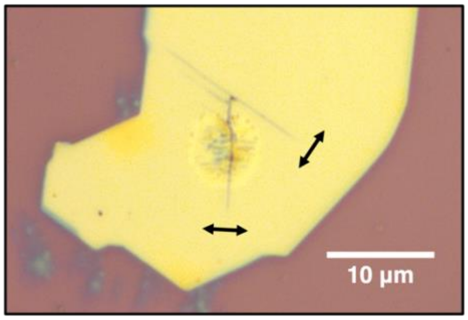

Fig. S5. From lines to shapes: Connecting zips at 60° angles.

Two zips are generated and elongated separately on a single flake with pump polarizations marked by the arrows. The individual zips, while not touching during the initial irradiation process, snap together to form 60° and 120° angles.

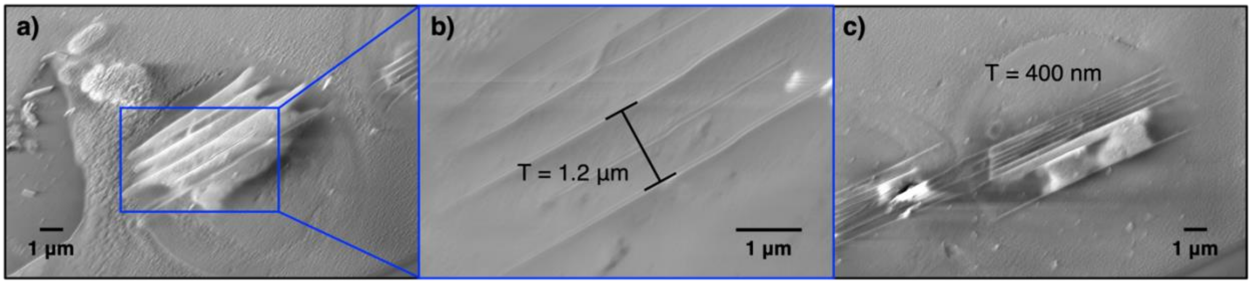

Fig. S6. SEM images of quasi-periodic gratings.

Quasi-periodic gratings on a 38-nm-thick hBN flake on SiO_2/Si. The zip width here is < 30 nm (below the SEM resolution in this configuration). **(A-B)** A quasi-periodic grating with a ~1.2 μm period. (The bubbles in (A) are imaging artifacts.) **(C)** Another grating on the same flake exhibiting a grating period of 400 nm. These "higher-order gratings" are less common.
